# “MS-Ready” structures for non-targeted high-resolution mass spectrometry screening studies

**DOI:** 10.1186/s13321-018-0299-2

**Published:** 2018-08-30

**Authors:** Andrew D. McEachran, Kamel Mansouri, Chris Grulke, Emma L. Schymanski, Christoph Ruttkies, Antony J. Williams

**Affiliations:** 1 Oak Ridge Institute for Science and Education (ORISE) Research Participation Program, U.S. Environmental Protection Agency, 109 T.W. Alexander Dr., Research Triangle Park, NC 27711 USA; 2 National Center for Computational Toxicology, Office of Research and Development, U.S. Environmental Protection Agency, Mail Drop D143-02, 109 T.W. Alexander Dr., Research Triangle Park, NC 27711 USA; 3 Present Address: Integrated Laboratory Systems, Inc., 601 Keystone Dr., Morrisville, NC 27650 USA; 4 Luxembourg Centre for Systems Biomedicine (LCSB), University of Luxembourg, 6, avenue du Swing, 4367 Belvaux, Luxembourg; 5 Department of Stress and Development Biology, Leibniz Institute of Plant Biochemistry (IPB), Weinberg 3, 06120 Halle (Saale), Germany

**Keywords:** High-resolution mass spectrometry (HRMS), Structure identification, Structure curation, Database searching

## Abstract

Chemical database searching has become a fixture in many non-targeted identification workflows based on high-resolution mass spectrometry (HRMS). However, the form of a chemical structure observed in HRMS does not always match the form stored in a database (e.g., the neutral form versus a salt; one component of a mixture rather than the mixture form used in a consumer product). Linking the form of a structure observed via HRMS to its related form(s) within a database will enable the return of all relevant variants of a structure, as well as the related metadata, in a single query. A Konstanz Information Miner (KNIME) workflow has been developed to produce structural representations observed using HRMS (“MS-Ready structures”) and links them to those stored in a database. These MS-Ready structures, and associated mappings to the full chemical representations, are surfaced via the US EPA’s Chemistry Dashboard (https://comptox.epa.gov/dashboard/). This article describes the workflow for the generation and linking of ~ 700,000 MS-Ready structures (derived from ~ 760,000 original structures) as well as download, search and export capabilities to serve structure identification using HRMS. The importance of this form of structural representation for HRMS is demonstrated with several examples, including integration with the in silico fragmentation software application MetFrag. The structures, search, download and export functionality are all available through the CompTox Chemistry Dashboard, while the MetFrag implementation can be viewed at https://msbi.ipb-halle.de/MetFragBeta/.

**Electronic supplementary material:**

The online version of this article (10.1186/s13321-018-0299-2) contains supplementary material, which is available to authorized users.

## Background

In recent years the use of high-resolution mass spectrometry (HRMS) instrumentation coupled to gas and liquid chromatography has become increasingly common in environmental, exposure and health sciences for the detection of small molecules such as metabolites, natural products and chemicals of concern [1–5]. Advances in instrumentation have led to faster acquisition times, lower limits of detection, and higher resolution, improving the rapid identification of chemicals of interest. However, the bottleneck of data processing has evolved to become the foremost challenge for non-targeted and suspect screening analyses (NTA and SSA, respectively) [1, 2, 6]. Workflows to address data processing can vary substantially between laboratories and depend on access to various software and programming capabilities. Common data processing workflows in NTA and SSA often utilize a combination of vendor-specific software, open source platforms, and in-house resources [1, 3, 7].

**Fig. 1:**
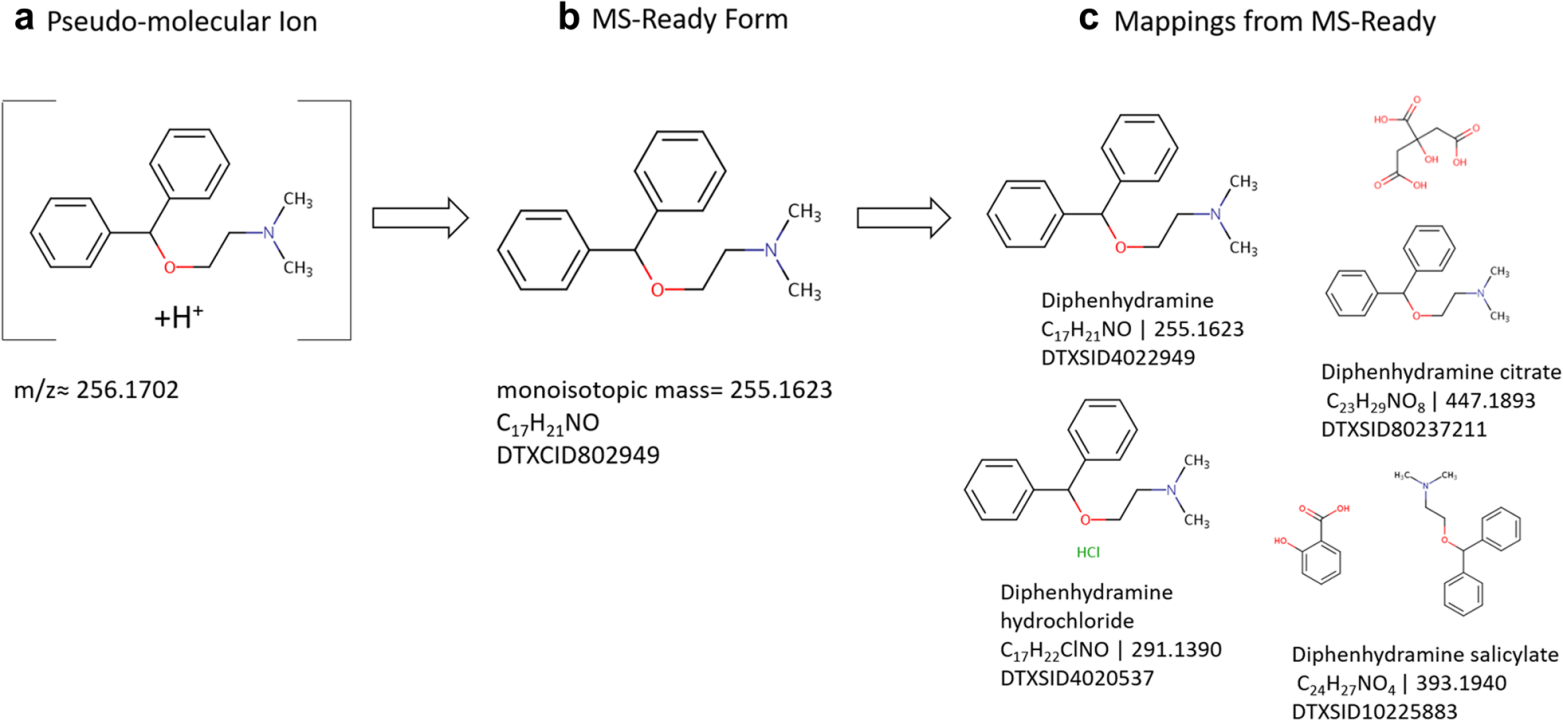
Using the example of the structure of diphenhydramine (DTXSID4022949 [17]): in HRMS, molecular features and associated ions are used to identify the pseudomolecular ion at a specific *m/z* (**a**). This information is then used to calculate the neutral monoisotopic mass and/or molecular formula (**b**). Both a neutral mass and formula can be searched in structure databases to retrieve matching candidate results (**c**). The MS-Ready form of a structure (**b** DTXCID802949 [18]) and the substance form(s) of a chemical (**c** DTXSID4022949 [17]; DTXSID80237211 [19]; DTXSID4020537 [20]; DTXSID10225883 [21]) are linked such that all can be retrieved in a single query with the EPA’s DSSTox database. DTXCID indicates the unique *chemical* identifier and DTXSID indicates the unique *substance* identifier, linked to metadata

In NTA the analyst generally uses peak-picking software to identify molecular features to find the (pseudo)molecular ion (*m/z*) along with associated isotopic peaks and calculate the neutral monoisotopic mass (Fig. 1a, b). Monoisotopic masses can be searched in structure databases to retrieve tentative candidates or can be used in combination with isotopic distributions and/or fragmentation data to arrive at a molecular formula(e) before candidate searching (Fig. 1c). Candidate selection often combines concepts such as database searching and data source ranking [7–9], spectral matching [10, 11] and retention time feasibility [7, 12–14] to identify the most probable structures, with database presence and metadata proving critical to success [7, 15]. When fragmentation information was combined with metadata and retention time information in MetFrag2.2, the number of correct identifications improved from 22% (105 of 473 correct) to 89% (420 of 473) on candidates retrieved from ChemSpider [16] using molecular formulae [7]. However, mixtures and salts (and thus their associated metadata) were excluded from candidate lists as these would not be observed at the calculated exact mass or formula used for searching. Yet, multi-component forms of a chemical (e.g., mixtures and salts, Fig. 1c) may contain the component observed via HRMS. Excluding these from database searches limits which substances can be identified by excluding variants of a structure and associated metadata.

Despite the prevalence of structure databases and online chemistry resources in NTA workflows, relatively little work has been done within the community to curate and standardize chemical structures in databases to optimize searching and identification with HRMS data [22, 23]. To maximize the search capabilities of structure databases, both the substance form, commonly represented by a structure (Fig. 1c), and the “MS-Ready” form (Fig. 1b) of the structure should be contained within databases and linked. When properly linked, both the observed form and variants of the structure observed via HRMS can be presented, thereby allowing the analyst to subsequently access metadata that may provide increased evidence in structure identification [5, 9, 15, 22, 24].

To link particular forms of a substance to their structure components (i.e., salts and mixtures) and their related MS-Ready forms, structure standardization is required. Various curation and standardization approaches are already defined in cheminformatics [25–28] and in use within the quantitative structure–activity relationship (QSAR) modeling community [27, 29]. QSAR modelers generally need desalted, neutralized, non-stereospecific structures, typically excluding inorganics and mixtures, to facilitate calculating molecular descriptors used in subsequent modeling approaches. Workflows describing the generation of QSAR-Ready structures have previously been published [27, 28, 30]. The requirements to produce MS-Ready structures are similar (vide infra), thus the processing rule set to produce QSAR-Ready files could be altered to provide an MS-Ready form of the data with a number of appropriate extensions. Hence, a previous QSAR-Ready structure preparation workflow [28, 30] was adapted to produce MS-Ready chemical structure forms that are amenable to structure identification using database searching. The resulting Konstanz Information Miner (KNIME) workflow, associated rule set and software processing module for the generation of MS-Ready structures are provided as an outcome of this work and available for download from a Github repository [31]. In addition, this workflow was used to generate MS-Ready forms (~ 700,000) for the ~ 760,000 chemicals substances in DSSTox [32] for access via the US EPA’s CompTox Chemistry Dashboard (hereafter “Dashboard”) [33]. The functionality in the Dashboard includes the ability to search, export and download MS-Ready structures. Several examples are provided to demonstrate the value of MS-Ready structures, including integration and demonstration of identification in NTA through the in silico fragmenter MetFrag [7]. Through accessibility to MS-Ready structures and the integration between the Dashboard and MetFrag, valuable resources to support structural identification of chemicals, now including mixtures and salts, are available to the community.

## Methods

### MS-Ready processing workflow

The MS-Ready processing workflow is an extension of the workflows described in detail by Mansouri et al. to curate and prepare QSAR-Ready structures for use in the development of prediction models [28, 30]. The related QSAR-Ready workflow is openly available on GitHub [34]. The free and open-source environment KNIME (Konstanz Information Miner) was used to design and implement the workflow [35]. Only free and open source KNIME nodes were used in the workflow. Cheminformatic steps were mainly performed using INDIGO nodes [36]. The nodes for each step were grouped into metanodes to ease readability and increase flexibility and future updates.

The MS-Ready workflow and transformation files are available on GitHub [31] and consisted of the following steps:

1. Consistency checking: file format, valence, and structural integrity.
2. Removal of inorganics and separation of mixtures into individual components.
3. Removal of salts and counterions (the salts list is available in Additional file 1).
4. Conversion of tautomers and mesomers to consistent representations. Examples include: nitro and azide mesomers, keto–enol tautomers, enamine–imine tautomers, enol-ketenes, etc. [37–39].
5. Neutralization of charged structures and removal of stereochemistry information.
6. Addition of explicit hydrogen atoms and aromatization of structures.
7. Removal of duplicates using InChIKey [40].

**Fig. 2:**
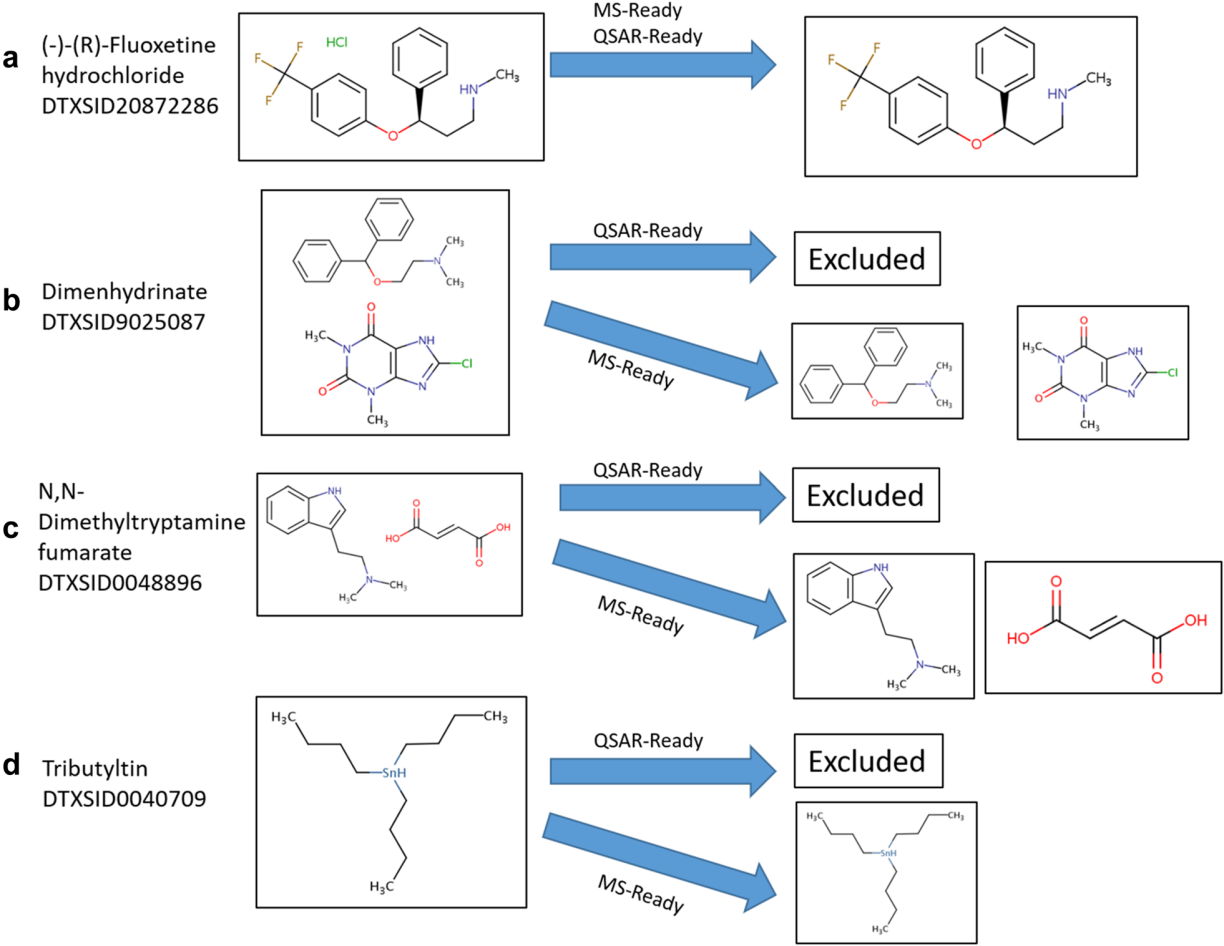
Original substances (left) and processed, linked chemical structures (right) indicating similarities and differences between the QSAR-Ready and MS-Ready workflows. **a** Salt and stereochemistry removed for both QSAR- and MS-Ready purposes; **b**, **c** mixtures separated and linkages retained for MS-Ready, discarded for QSAR-Ready; **d** organometallics with metal–carbon bonds retained in MS-Ready, discarded in QSAR-Ready. The identities of the associated MS-Ready structures are visible in the “Linked Substances” tab of individual substance records in the Dashboard

Differences between the QSAR-Ready and MS-Ready workflows exist primarily in the handling of salts and counterions, chemical mixtures, metals, and organometallics (Fig. 2). For the generation of both QSAR and MS-Ready structures, salts and solvents are separated and removed from mixtures via an exclusion list (Fig. 2a). The exclusion list used during QSAR-Ready structure preparation (189 structures, SDF file provided as Additional file 2) was substantially reduced for MS-Ready structures (32 structures, SDF file provided as Additional file 1), allowing a greater number of secondary components that are observable in MS to be retained and linked to the original substances via MS-Ready forms (e.g., benzoate, fumarate, citrate). For MS-Ready structures, all records still containing multiple components were separated out, deduplicated if necessary, and retained, with all components linked to the original substance (Fig. 2b, c). For the QSAR-Ready workflow, in contrast, chemical mixtures are excluded due to the complexity merging activity estimates for components of the mixture (Fig. 2b, c). The MS-Ready workflow retains organometallics containing covalent metal–carbon bonds within the chemical structure while the QSAR-Ready workflow does not (Fig. 2d), primarily because most descriptor packages used for QSAR modeling cannot handle organometallic compounds. However, users of MS-Ready structures for environmental and exposure NTA applications need to include substances such as organomercury and organotin compounds, due to their toxicity and use as, for example, fungicides and antifouling agents.

### Mapping MS-Ready structures to substances

For the purpose of structure identification using the Dashboard, MS-Ready structures must be mapped to the associated chemical substances in the underlying DSSTox database [32]. Chemical substances within DSSTox are identified by unique DTXSIDs (DSSTox Substance Identifiers) and can denote a mixture, polymer or single chemical while DTXCIDs (DSSTox Chemical Identifier) are unique chemical structure identifiers. A structure-data file (SDF) of all chemical structures (DTXCIDs) associated with substances (DTXSIDs) was exported and passed through the MS-Ready preparation workflow. The resulting MS-Ready structures were then loaded back into the DSSTox structure table, omitting duplicate structures as identified by standard InChIKey [40] generated using the JChem Java API [41]. Mappings between the original DSSTox structure and its MS-Ready form was stored in a structure relationship mapping table.

### Accessibility to MS-Ready results

**Fig. 3:**
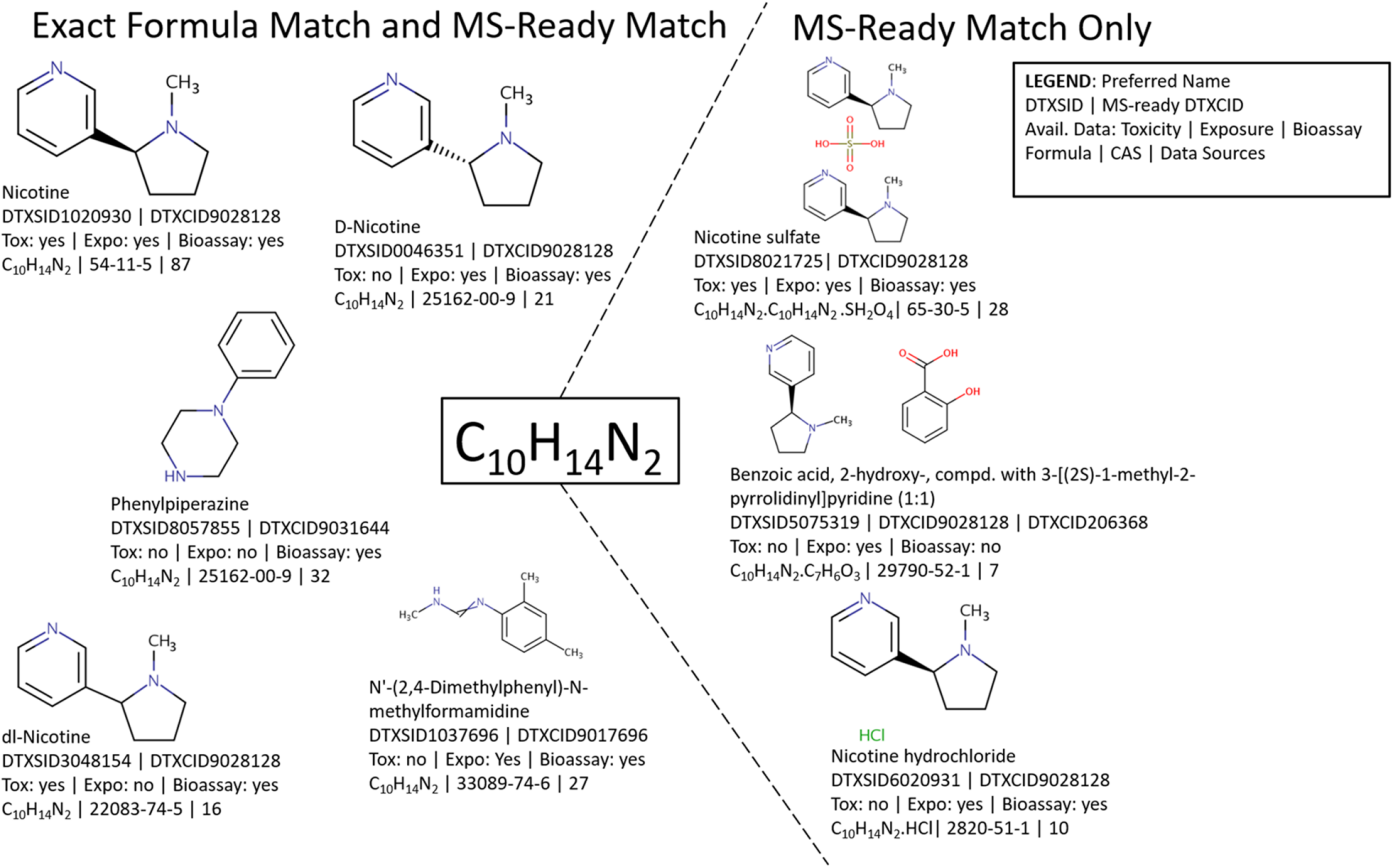
Results of both an exact formula (left) and MS-Ready formula search (all) demonstrated using the molecular formula of nicotine (C_10_H_14_N_2_), top left. A search of C_10_H_14_N_2_ using the MS-Ready search functionality [45] retrieves all 8 substances while an exact formula search [46] retrieves only the 5 on the left. The MS-Ready DTXCID representing the chemical structure of nicotine is present in 6 of the 8 example substances (DTXCID9028128). Metadata such as toxicity, exposure, and bioactivity data vary for all results. Accessing the data for the mixtures, salts, etc. is unachievable in a single search without linking through the MS-Ready form. Figure based on the concept illustrated by Schymanski and Williams (2017), with permission [22]

Once mapped within the database, functionality to support searching based on MS-Ready structures was incorporated into the Dashboard [33] to support mass spectrometry-based NTA and SSA. MS-Ready structures can be searched using the Advanced Search page based on a single molecular formula [42] or can be searched in batch mode (i.e., 1–100 s of masses or formulae at a time) in the Batch Search interface [43]. The Batch Search interface allows for MS-Ready structure searching of both molecular formulae and monoisotopic masses. As the form of a chemical structure observed via HRMS is linked to all substances containing the structure (e.g., the neutral form, all salt forms, mixtures), when a molecular formula or monoisotopic mass is searched using MS-Ready structures, both single component and multi-component substances can be returned. This is distinct from an exact formula search whereby results returned match the input formula exactly (e.g., excluding mixtures where only a component matches that given formula). Figure 3 demonstrates the difference between an exact formula search (returning candidates to the left of the figure) and an MS-Ready search (which returns all candidates shown in the figure). Both exact formula and MS-Ready formula searches can be conducted within the Advanced Search and Batch Search pages of the Dashboard. Screenshots of the search interfaces and resulting file are provided in Additional file 3: Figs. S1–S4. Users can download the results with export options including SMILES and the identifiers that correspond with the substance (CASRN, preferred name, synonyms), chemical and MS-Ready forms. Column headers specify the individual component structure (DTXCID) that was matched to the input as well as the mapped substance (DTXSID) and substance-associated data (Additional file 4: Tables S1 and S2). Additionally, users can include other data from the Dashboard export pane that is relevant to their needs (e.g., exposure data, bioactivity data, property predictions, presence in lists). This MS-Ready batch search option is designed to enable candidate retrieval through searching large numbers of suspect formulae and masses (Additional file 4: Table S2) [9]. By selecting the “MetFrag Input File” option in the Batch search, users can generate a file (including any selected metadata) containing all relevant structural information required for MetFrag to upload and process MS-Ready structures correctly (see below).

An MS-Ready file generated from all chemical structures contained within the DSSTox database is available for download [44]. With this file, users may create their own databases to incorporate into instrument software for screening.

### Integration with MetFrag

The export option (“MetFrag Input File (Beta)” under *Metadata*) was added to the Batch Search page to create an MS-Ready export file suitable for direct import into the in silico fragmenter MetFrag [7, 47]. As outlined above, mixtures and salts are excluded in MetFrag by default. However, through the MS-Ready export file, MetFrag can now process the component of the mixture observed at the given input formula (i.e., the MS-Ready form) and retain the metadata and identifiers associated with the substance form (mixture, salt, original substance). Column headers in the Dashboard export were elaborated to distinguish the individual component structure (DTXCID) and associated data from data related to the substance (DTXSID). By default, the export file from the Dashboard contains the fields: INPUT; FOUND_BY; DTXCID_INDIVIDUAL_COMPONENT; FORMULA_INDIVIDUAL_COMPONENT; SMILES_INDIVIDUAL_COMPONENT; MAPPED_DTXSID; PREFERRED_NAME_DTXSID; CASRN_DTXSID; FORMULA_MAPPED_DTXSID; SMILES_MAPPED_DTXSID; MS_READY_SMILES; INCHI_STRING_DTXCID; INCHIKEY_DTXCID; MONOISOTOPIC_MASS_DTXCID (Additional file 4: Table S3). Users can select any other additional data fields on the Batch Search page to include in the MetFrag scoring (details below). In this export file, MetFrag treats the “DTXSID” (substance identifier) field as the identifier, but takes the structural information (formula, mass, SMILES, InChI, InChIKey) from the fields denoted with DTXCID (which corresponds with the structure observed in MS). The other fields are included in the export file so that users can display the mixture or components. Any additional data fields that contain numeric data are automatically imported by MetFrag and included as an additional “Database scoring term” in the “Candidate filter & Score Settings” tab (Additional file 5: Figure S5).

By default, MetFrag groups all candidates with the same InChIKey first block, reporting only results from the highest scoring member of the group. However, the MS-Ready search involves components of mixtures, where individual components are often also in the Dashboard and contain different metadata. Merging these by the component InChIKey would result in a loss of the metadata obtained from the Dashboard search. To retain all candidates, the “Group candidates” option in the “Fragmentation Settings and Processing” tab should be deselected. Even if candidates are grouped, all substance identifiers within a group are still displayed and hyperlinked to the Dashboard (see Additional file 5: Fig. S6).

### MetFrag example calculations

To demonstrate the workflow, the results of an MS-Ready formula search for C_9_H_16_ClN_5_ (terbutylazine) and C_7_H_12_ClN_5_ (desethylterbutylazine) were exported as.csv for import into MetFrag. The.csv file was imported into the MetFragBeta web interface [47] and the candidates were selected by molecular formula. Experimental fragmentation data were retrieved from the European MassBank [48] to conduct the queries in MetFrag. Spectral data for terbutylazine (DTXSID4027608 [49]) was collected from record EA028406 [50], recorded at collision energy HCD 75 (higher-energy collisional dissociation) and resolution 7500 (MS/MS) on an LTQ Orbitrap XL (at Eawag, Switzerland). Spectral data for desethylterbutylazine (DTXSID80184211) was also retrieved from MassBank, record EA067106 [51], likewise a MS/MS spectrum measured at HCD 75 and R = 7500 on the LTQ Orbitrap XL at Eawag. Metadata from the Dashboard that were included as scoring terms were: Data Sources, PubMed Reference Count, ToxCast % active and the presence in two lists: Norman Priority [52] and STOFF-IDENT [53]. The use of data sources in the Dashboard for identification of unknowns has been documented [9] and combined ranking schemes using multiple data streams and database presence are being optimized in current research. The metadata selected here should not be considered finalized scoring parameters but primarily to demonstrate functionality. The fragmentation settings were Mzppm = 5, Mzabs = 0.001, Mode = [M+H]^+^, Tree depth = 2, Group candidates = deselected. In addition to the Dashboard scoring, the MetFrag Scoring Term “Exact Spectral Similarity (MoNA)” was activated [54]. On the MetFrag web interface, the combination of the regular MetFrag Fragmenter score (ranging from 0 to 1), the spectral similarity term (also ranging from 0 to 1) and each metadata field creates an additive score, with the maximum determined by the number of metadata fields selected. For example, the MetFrag Fragmenter score, spectral similarity score and 5 metadata categories mentioned here will result in a maximum score of 7, where the scores for each individual category are automatically scaled between 0 and 1 based on maximum values (no data gives score = 0). While it is possible to perform more sophisticated scoring via the command line version, this is beyond the scope of the current article—the work presented here is intended to demonstrate the potential for the MS-Ready approach to support identification efforts. Additional examples not described in the text are provided in the Additional file 5 (Figures S7–S8 for C_10_H_14_N_2_, the formula of nicotine, and C_17_H_21_NO, the formula of diphenhydramine, respectively).

## Results and discussion

### Linking metadata via MS-Ready structures

**Fig. 4:**
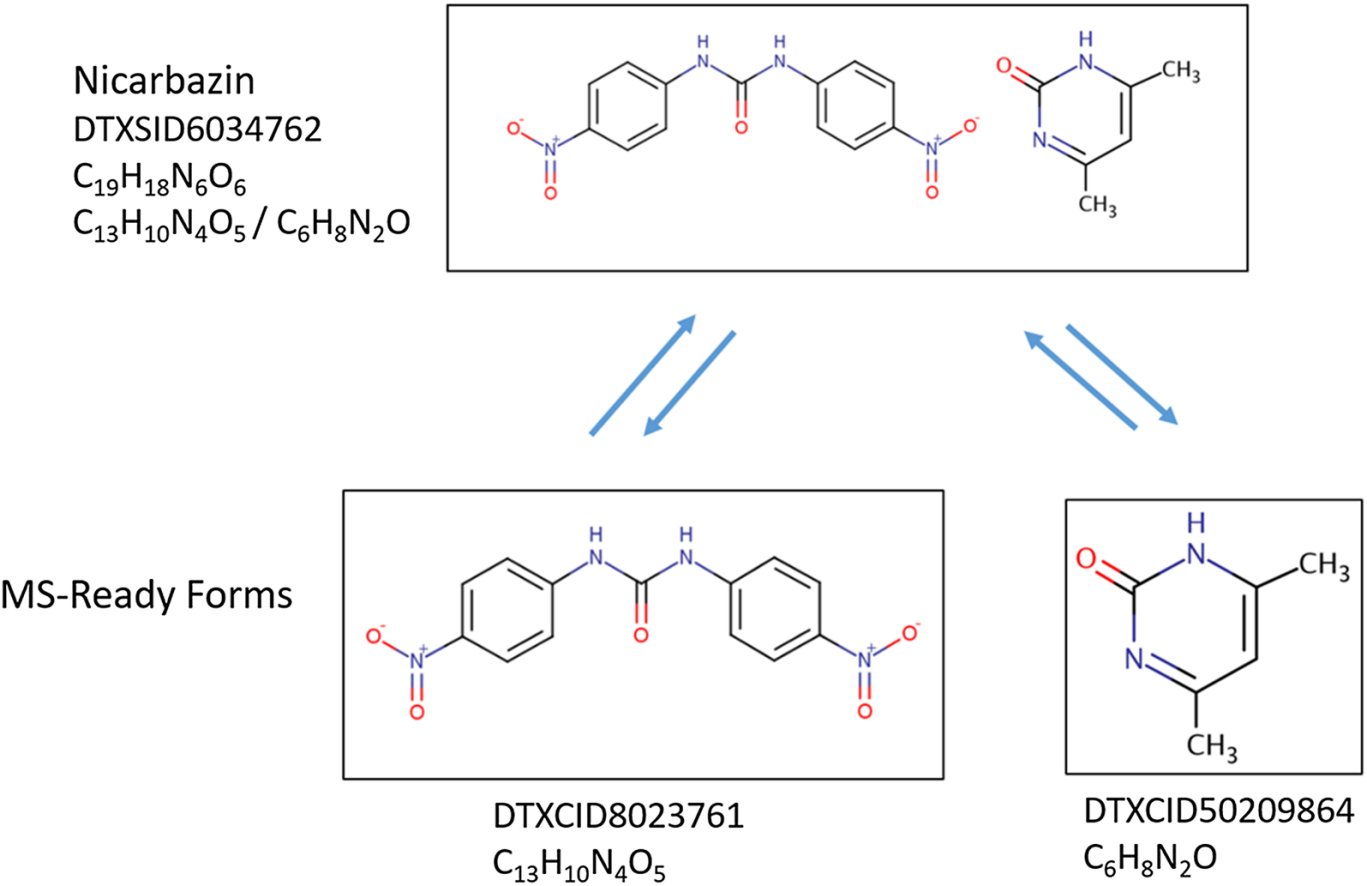
The substance Nicarbazin (DTXSID6034762) and its two components (DTXCID8023761; DTXCID50209864), separated as a result of the MS-Ready workflow. The MS-Ready forms are linked to the source substance and enable retrieval of associated structures and metadata through a single database query

It has been demonstrated that data sources and other metadata linked to chemical structures improve identification of unknowns [7, 15, 55]. Substances in the Dashboard contain different linked metadata [22], making access to all forms of a chemical structure important for identification (Fig. 3). Beyond data sources alone, chemical functional use and product occurrence data [56, 57] are metadata that can help analysts arrive at the source of a chemical in a sample through mapping via MS-Ready structures. Nicarbazin (DTXSID6034762, C_19_H_18_N_6_O_6_ [58]), a coccidiostat used in poultry production, is a two component chemical (with the associated formulae for the two separate structures being C_13_H_10_N_4_O_5_ and C_6_H_8_N_2_O) whose components would dissociate in the environment, leading to the observation of individual components only via HRMS. Neither of the single components has known commercial uses (yet) that would result in environmental occurrence. By mapping the two observable components to the source substance, the analyst is potentially able to identify the substance likely used in commerce with an observed formula search (Fig. 4), thereby improving exposure characterization where accurate identification of source substances is critical. Furthermore, the presence of one part of a component may indicate the presence of the other component in the sample, triggering further identifications. Informing the analyst of the most likely substance, rather than just the chemical structure identified by HRMS, may allow decision makers and risk assessors the ability to link chemical identifications and substances. The application of this during candidate selection in non-target screening is discussed further below.

### Non-target collaborative trials

**Fig. 5:**
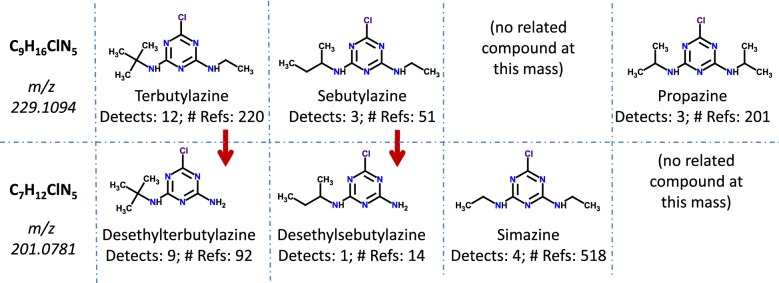
Tentative annotations of *m/z* 229.1094 (top) and *m/z* 201.0781 provided by NORMAN Collaborative Trial participants. Number of detects indicates the number of participants in the collaborative trial who provided the structural annotation of the selected compound. Reference data is from ChemSpider. Source data and figure modified from Schymanski et al. [2]

In 2013, the NORMAN Network coordinated a collaborative non-targeted screening trial on a river water sample [2]. Several examples from this trial indicated the need for improved curation of chemical structures as well as better metadata linkage across substances in a sample during non-targeted screening. Participants reported, for instance, mass matches to the salt form of a substance in a suspect list (e.g., tris[4-(diethylamino)phenyl]methylium acetate, C_31_H_42_N_3_.C_2_H_3_O_2_ reported at *m/z* 516.3565 by one participant, which could not be observed in the sample as the acetate would dissociate). Using MS-Ready structures can reduce errors associated with identifying salt forms by searching at the single component level and returning mapped substances. The complex nature of considering metadata and sample context in non-target identification is further demonstrated with the tentative annotations provided for the masses *m/z *= 229.1094 and 201.0781 (see Fig. 5, adapted from Fig. 2 in [2]). For *m/z *= 229.1094, most participants provided the tentative annotation for terbutylazine (DTXSID4027608, which many participants had as a target analyte). Propazine (DTXSID3021196) is not approved for use in Europe and should not be detected in typical environmental samples, yet it was still reported three times due to the high reference count. For *m/z *= 201.0781, the presence of terbutylazine provides strong evidence to support the tentative annotation of desethylterbutylazine (DTXSID80184211), although many participants reported simazine (DTXSID4021268) due to its higher reference count (Fig. 5). Simazine and desethylterbutylazine (with the often co-eluting desethylsebutylazine, DTXSID20407557) can often be distinguished using fragmentation information.

The EPA’s Non-Targeted Analysis Collaborative Trial (ENTACT) was initiated following the NORMAN collaborative trial [2]. ENTACT is an inter-laboratory trial where participating laboratories and institutions were provided blinded chemical mixtures and environmental samples for NTA and SSA [59, 60]. The blinded chemical mixtures included several multi-component substances that could be either mismatched or unidentified without a linkage between the MS-Ready form of a chemical structure and its multi-component form (e.g., chemical mixtures, salts). For the purposes of ENTACT, identification of the original substances added to the mixtures is critical to the trial evaluation. Methapyrilene fumarate (DTXSID0047404 [61]), for example, is a mixture of two chemical components (in a 3:2 ratio) that would be observed separately (DTXCID003278 [62]; DTXCID8028133 [63]), while raloxifene hydrochloride (DTXSID1034181 [64]) is a substance containing a hydrochloride salt that would be matched incorrectly from MS data without the appropriate standardization and linking. Linking the MS-Ready forms of these chemicals to the substance forms facilitates identification by including all variants in the search results with associated metadata. For example, blinded analysis of one of the ENTACT mixtures resulted in the observation of *m/z *= 262.1385 in ESI+ (Sobus et al. submitted for publication). With this exact mass and associated isotopic peaks, the formula C_14_H_19_N_3_S was generated. When the formula was searched in the Dashboard (C_14_H_19_N_3_S [65]) the results included both the single component methapyrilene (DTXSID2023278 [66]) and the multi-component methapyrilene fumarate (DTXSID0047404 [61]) in the top 5 results as ranked by data source count. An exact formula search would not have returned the substance originally added to the ENTACT mixture, which was in fact methapyrilene fumarate. The MS-Ready search in the Dashboard and the linkages are especially beneficial when the structures identified by HRMS differ from the form of the substance initially contained within the mixture (e.g., Fig. 4). In addition to the Dashboard MS-Ready functionality in the user interface, files containing MS-Ready forms of the chemical structures, mapped to the original chemical substances contained within the mixtures, were provided to the participants as part of ENTACT and are available via the Dashboard as an Excel spreadsheet [44].

### Enhanced searching: an example with perfluorinated chemicals

**Fig. 6:**
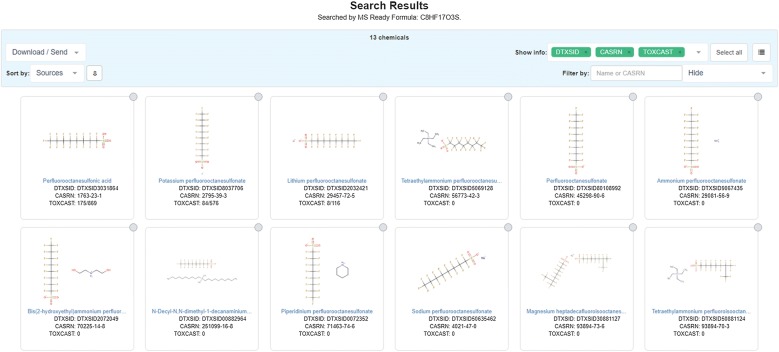
Partial results from an MS-Ready formula search of the neutral formula of PFOS (C_8_HF_17_O_3_S) in the Dashboard [78]. The neutral acid, the sulfonate (C_8_F_17_O_3_S^−^), and multiple salts and mixtures containing PFOS are returned in the results list

With an increasing focus on perfluorinated chemicals and their effects on the environment and public health [67–71], it is not only important to be able to accurately identify perfluorinated structures in environmental samples but also to identify the potential sources of the contaminant for exposure characterization. Perfluorinated chemicals also present a challenge for NTA, as the presence of monoisotopic fluorine renders calculation of possible molecular formulae very challenging [5, 72]. As a result, SSA and compound database searching is advantageous to finding these compounds. Perfluorosulfonic acids (e.g., PFOS, DTXSID3031864 [73]), perfluorocarboxylic acids (e.g., PFOA, DTXSID8031865 [74]), and other similar structures are thought to occur in the environment as anions [67]. Hence, these structures are often reported in the literature as anions, but have also been reported as neutral acids. In chemical databases these structures can be represented in their neutral forms, as a part of chemical mixtures, and as multi-component salts (e.g., PFOS-K, DTXSID8037706 [75]), representing the myriad of chemical forms available in commerce (see the linked MS-Ready substances for PFOS currently in the Dashboard [76]). PFOS would generally be observed by an analyst via HRMS as a negatively charged *m/z* feature (C_8_F_17_O_3_S^−^), and when a neutral monoisotopic mass is calculated, the analyst is likely to arrive at the molecular formula of the neutral acid form of PFOS (C_8_HF_17_O_3_S). Searching the neutral formula of PFOS (C_8_HF_17_O_3_S) in the Dashboard MS-Ready Batch Search option returns the neutral acid, the sulfonate (C_8_F_17_O_3_S^−^), and multiple salts and mixtures containing PFOS in the results list (Fig. 6). These results include the neutral form and the substance forms thought to occur in the environment and used in consumer products/commerce, along with associated metadata. Many forms of PFOS may be contained in other public databases, and other strategies have been developed to counteract the anion/neutral form issue during compound searching (e.g., UC2 by Sakurai et al. [77]). The current MS-Ready functionality in the Dashboard provides mappings to multiple forms of chemicals related via their “MS-Ready” form in a single search, improving researchers’ ability to identify sources and improve exposure characterization with increased coverage and access to metadata.

### Non-target identification: in silico methods and candidate searching

In this section two examples from the NORMAN Collaborative Trial (Fig. 5) are used to show how the MS-Ready form of a mixture will help analysts combine MS evidence (such as fragments) with mixture metadata for candidate screening in NTA. By crosslinking with the MS-Ready form through the export format described above, the candidates can be processed using MS-Ready structures, with metadata from the mixture in MetFrag. As described in the Methods (*MetFrag Example Calculations*), two MetFrag scoring terms plus five metadata terms were used, which would result in a maximum possible score of 7 for candidates in each example.

**Fig. 7:**
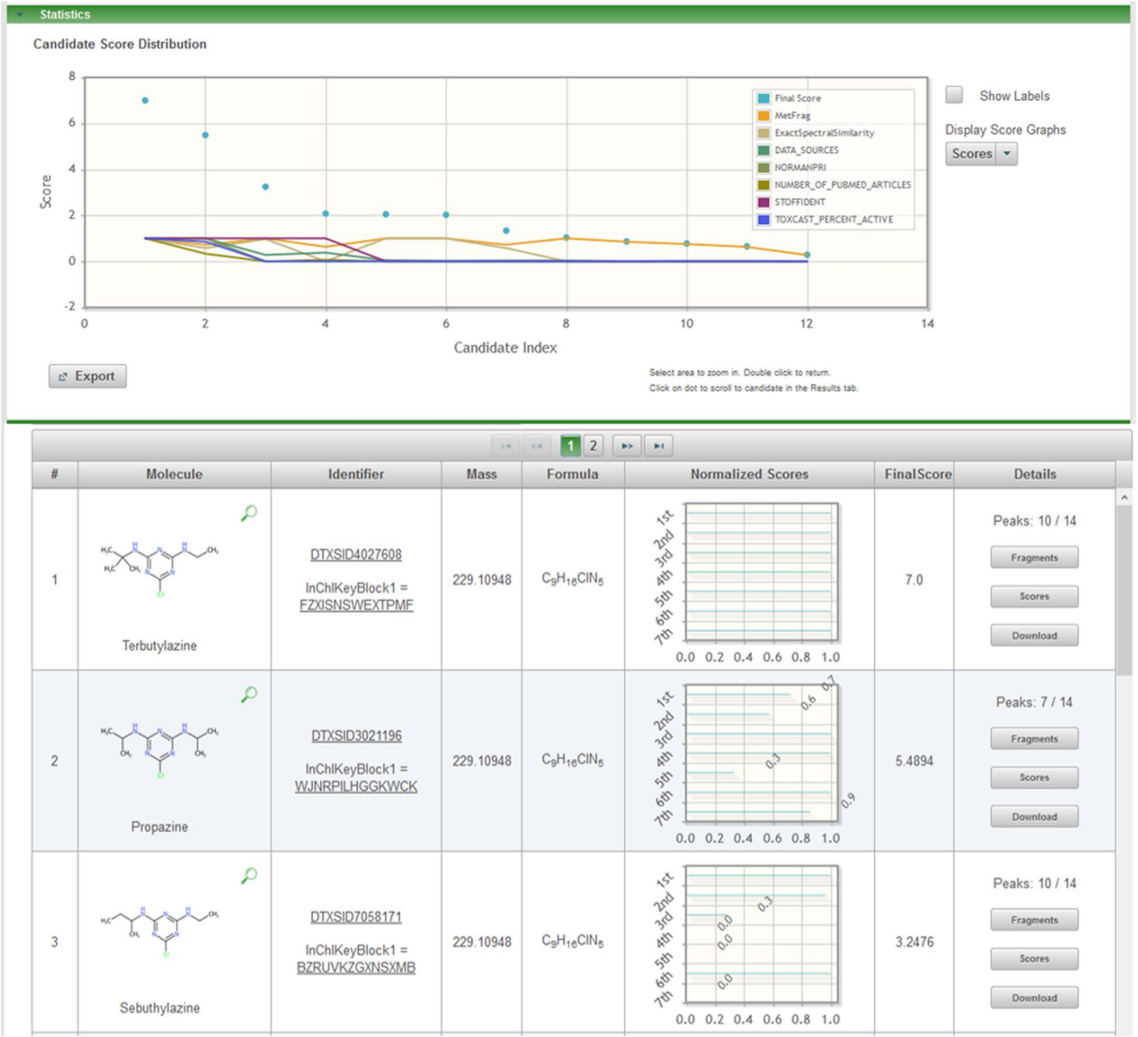
MetFrag combined results (top) and results for the top 3 candidates (bottom) retrieved with the MS-Ready search for C_9_H_16_ClN_5_. The score categories are (1st to 7th): MetFrag Fragmentation, Exact Spectral Similarity, Data Sources, Presence in NORMAN Priority list, Number of PubMed Articles, Presence in STOFF-IDENT, and Percent Active ToxCast Assays. Terbutylazine had the highest score, above propazine. Sebutylazine (which, if present, often co-elutes with terbutylazine in common NTA methods) has a lower score due to fewer metadata values (absent from NORMAN list and no ToxCast bioassay data)

**Fig. 8:**
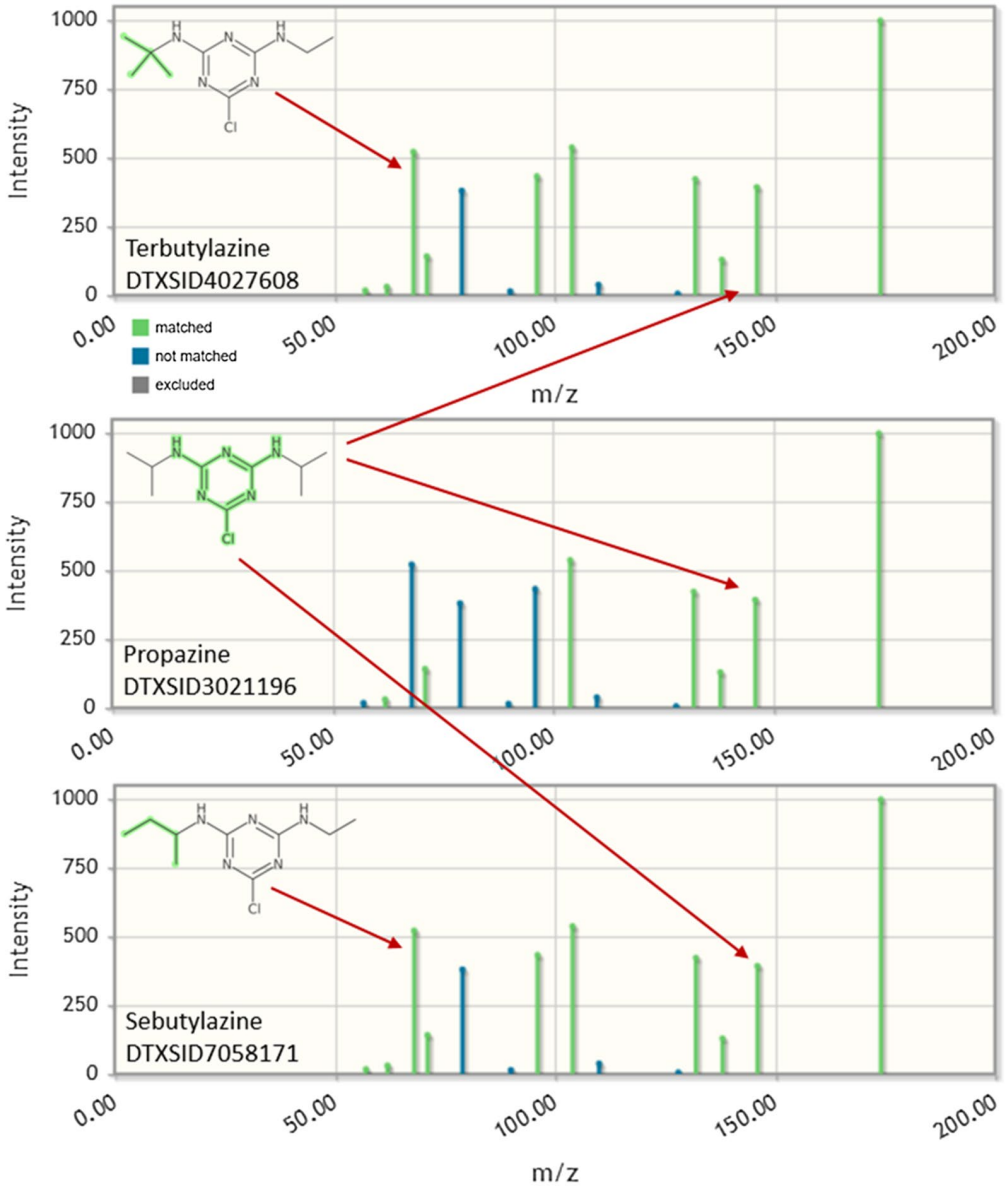
MetFrag Fragmentation results for the top three candidates retrieved with the MS-Ready search for C_9_H_16_ClN_5_. Terbutylazine (top) has the highest score and includes the C_4_H_9_^+^ fragment at *m/z* = 57.0698 indicating the presence of a butyl substituent, absent from propazine (middle)

The results for the top three candidates from the first example, C_9_H_16_ClN_5_, using fragmentation data from terbutylazine are shown in Fig. 7. This demonstrates how the combination of fragmentation prediction, MS/MS library matching, and metadata supports the annotation of terbutylazine (MetFrag Score 7.0, including an exact spectral match of 1.0 from MoNA—i.e., a Level 2a identification [24]) above propazine (MetFrag Score 5.5, exact spectral match 0.5774, i.e., a poor match). The presence of the C_4_H_9_^+^ fragment at *m/z* = 57.0698, explained by MetFrag, indicates the presence of a butyl substituent, absent from propazine (Fig. 8). Sebutylazine, the third candidate, has a much lower score due to fewer metadata (see Fig. 7), although the fragmentation data is very similar to terbutylazine (Fig. 8).

**Fig. 9:**
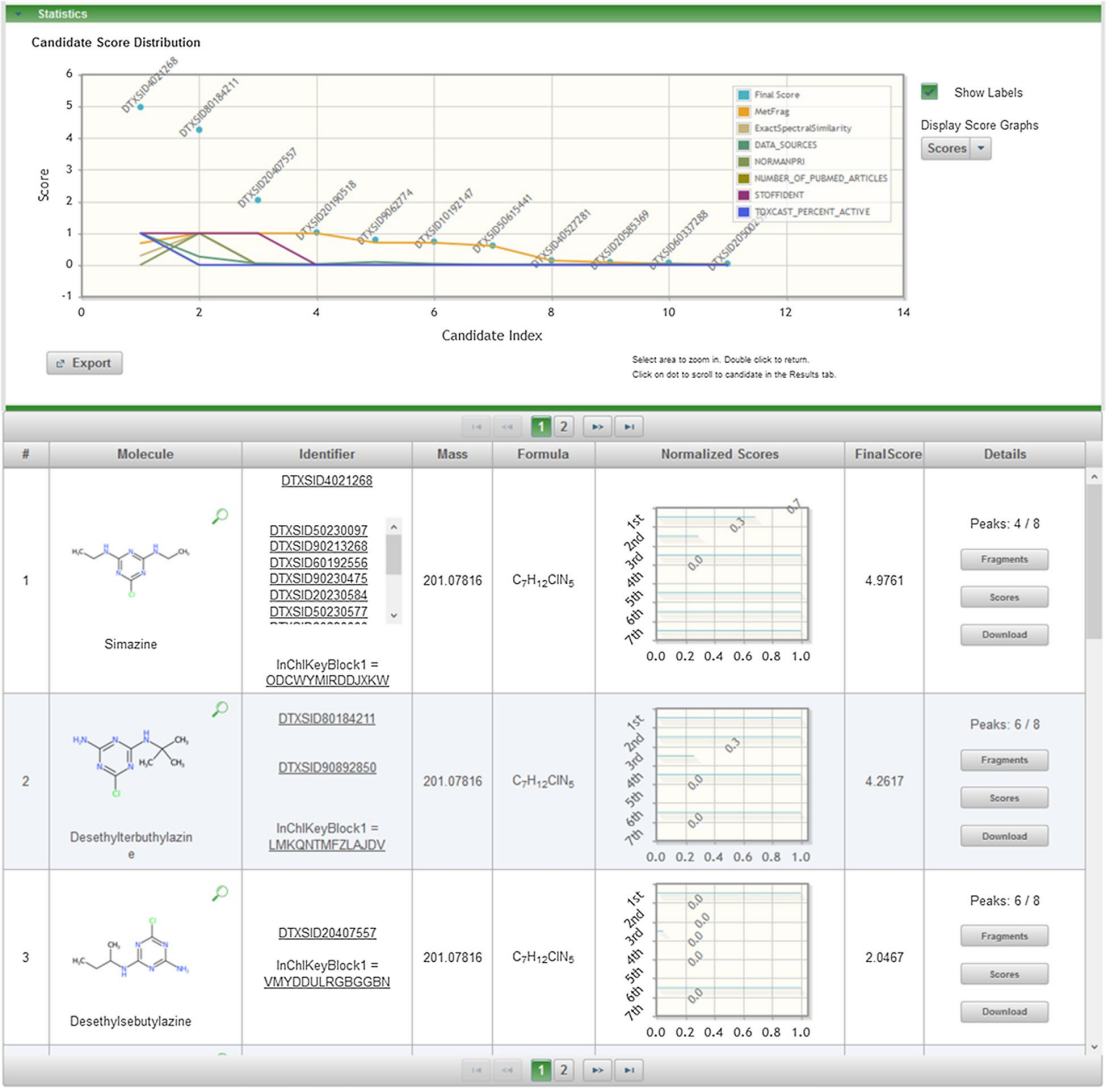
MetFrag combined results (top) and results for the top 3 candidates retrieved with the MS-Ready search for C_7_H_12_ClN_5_ (as displayed in the web interface). The score categories are (1st to 7th): MetFrag Fragmentation, Exact Spectral Similarity, Data Sources, Presence in NORMAN Priority list, Number of PubMed Articles, Presence in STOFF-IDENT, and Percent Active ToxCast Assays. Candidate merging was activated and the 10 forms of simazine have been merged into one result (with metadata from the highest scoring entry)

The second example, the MS-Ready search for C_7_H_12_ClN_5_ with the spectral data of desethylterbutylazine, was run with the same settings, but with the candidate grouping activated. The top three candidates from the MetFrag web interface [47] are given in Fig. 9 and detailed scores are provided in Additional file 5: Table S4. The top-ranked candidate with the selected metadata and default scoring is simazine (Score 4.98 of maximum 7.0). It is also clear from the numerous DTXSID values displayed in the “Identifier” column for simazine that there are many substances (mixtures, salts) in the Dashboard that contain simazine as one component (11 of the 21 candidates returned in the MS-Ready search). Desethylterbutylazine is in second place with a score of 4.26. Additional file 5: Figs. S7 and S8 show MetFrag results for additional searches correctly placing nicotine (DTXSID1020930) and diphenhydramine (DTXSID4022949) as the top result, respectively, with the same metadata options included and candidate grouping activated.

The example in Fig. 9 demonstrates how users must think critically about the impact of the metadata on the results. While simazine (Score 4.98) outranks desethylterbutylazine (Score 4.26), closer inspection reveals this result is due to metadata score influence. The experimental data (fragmentation prediction, peaks explained, spectral similarity, exact spectral similarity) matches better for desethylterbutylazine (6/8 peaks explained and scores close to or equal to 1 for the other experimental fields) than for simazine. Desethylterbutylazine does not have a ToxCast Bioassay score and has no PubMed references, resulting in two zero scores, while simazine has a score of 1 for both of these metadata categories. Furthermore, while the MetFrag website [47] provides users with a convenient interface to score with a tick-box, users must be aware of the limitations inherent in providing a convenient interface. The data in each external category is imported and scaled between 0 and 1 using the minimum and maximum values, which is not meaningful for all metadata categories (such as predicted properties). Note that it is possible to adjust the weighting and relative contributions of the scores by adjusting the bars on the “Weights” field at the top of the results page (once candidates are processed), while additional scoring possibilities are available via the command line version.

### Improvements and future work

Beyond access to structures and workflows via the Dashboard, future functionality of the Dashboard will allow for users to upload structure files and receive back the MS-Ready version of the structures of interest, increasing standardization across database searching and compound identification. Alterations to the output format (as described in the Methods) will enable other in silico fragmentation and compound identification tools, methods, and software to use the work described here. Further flexibility in file formats will be implemented to achieve broader usability. As with any chemical structure standardization workflow, algorithms are modified to deal with edge cases as they are identified. As the database content continues to expand, the algorithm is improved as failures are identified. While the MS-Ready approach may lead to potentially confusing results sets containing structures with different formulae and masses than specified in the original search parameters, communication, education, and transparency within the Dashboard interface, download files, and publications will serve to clarify and provide guidance. Finally, to facilitate access to the underlying data for structure identification on the broadest scale, an application programming interface (API) and associated web services to allow instrument software integration is forthcoming. These will enable access via applications such as Python, R, and Matlab to facilitate integration of Dashboard data into user-specific applications.

## Conclusions

Database searching is a vital part of NTA and SSA workflows. The accurate mapping of MS-Ready structures to chemical substances improves accessibility to structure metadata and improves searching of the represented chemical space. By providing access to MS-Ready data from DSSTox, both via the Dashboard and as downloadable datasets, users of HRMS instrumentation who perform NTA/SSA experiments will benefit from this approach as an enhancement to other online databases that do not support MS-Ready structural forms. The integration into the in silico fragmenter MetFrag lets users further explore the use of this approach in identification of unknowns. The openly available workflow for generation of MS-Ready structures allows others to process their own data for preparation of MS-Ready data files and extend the data handling to account for errors and specific cases that we have not yet identified.

## Additional files

**Additional file 1.** MS-Ready exclusion list.

**Additional file 2.** QSAR-Ready exclusion list.

**Additional file 3.** CompTox Chemistry Dashboard search interfaces (**Figures S1–S4**).

**Additional file 4.** Download file column header descriptions and example output files for MS-Ready and MetFrag Input File batch searches (**Tables S1–S3**).

**Additional file 5.** Additional MetFrag results and data (**Figures S5–S8**, **Table S4**).

## Abbreviations

HRMS: high-resolution mass spectrometry
DSSTox: distributed structure-searchable toxicity
ENTACT: EPA’s non-targeted analysis collaborative trial
QSAR: quantitative structure activity relationship
NTA: non-targeted analysis
SSA: suspect screening analysis
