# Supplementary material for: “MS-Ready” structures for non-targeted high-resolution mass spectrometry screening studies
Source: J Cheminform. 2018 Aug 30;10:45. doi: 10.1186/s13321-018-0299-2 (PMC6117229; doi:10.1186/s13321-018-0299-2)
Supplement: Supplementary file 3 — Additional file 3. CompTox Chemistry Dashboard search interfaces (Figures S1–S4). [file 13321_2018_299_MOESM3_ESM.docx]

Additional File 3, 4 Figures

“MS-Ready” structures for non-targeted high-resolution mass spectrometry screening studies

Andrew D. McEachran^1,2*^, Kamel Mansouri^1,2,3^, Chris Grulke^2^, Emma L. Schymanski^4^, Christoph Ruttkies^5^ and Antony J. Williams^2*^

^1^Oak Ridge Institute for Science and Education (ORISE) Research Participation Program, U.S. Environmental Protection Agency, 109 T.W. Alexander Dr., Research Triangle Park, NC 27711 USA

^2^National Center for Computational Toxicology, Office of Research and Development, U.S. Environmental Protection Agency, 109 T.W. Alexander Dr., Research Triangle Park, NC 27711 USA

^3^ Current address: Integrated Laboratory Systems, Inc., 601 Keystone Dr, Morrisville, NC 27650, USA

^4^Luxembourg Centre for Systems Biomedicine (LCSB), University of Luxembourg, 6, avenue du Swing, L-4367 Belvaux, Luxembourg.

^5^Department of Stress and Development Biology, Leibniz Institute of Plant Biochemistry (IPB), Weinberg 3, 06120 Halle (Saale), Germany.

Email addresses:

Kamel Mansouri: [kamel.mansouri@nih.gov](mailto:kamel.mansouri@nih.gov)

Chris Grulke: [grulke.chris@epa.gov](mailto:grulke.chris@epa.gov)

Emma L. Schymanski: [emma.schymanski@uni.lu](mailto:emma.schymanski@uni.lu)

Christoph Ruttkies: [christoph.ruttkies@ipb-halle.de](mailto:christoph.ruttkies@ipb-halle.de)

Andrew D. McEachran Antony J. Williams

[mceachran.andrew@epa.gov](mailto:mceachran.andrew@epa.gov) [williams.antony@epa.gov](mailto:williams.antony@epa.gov)

Mail Drop D143-02 Mail Drop D143-02

109 T.W. Alexander Dr. 109 T.W. Alexander Dr.

Research Triangle Park, NC 27711 USA Research Triangle Park, NC 27711 USA

Phone: 1-919-541-3001 Phone: 1-919-541-1033


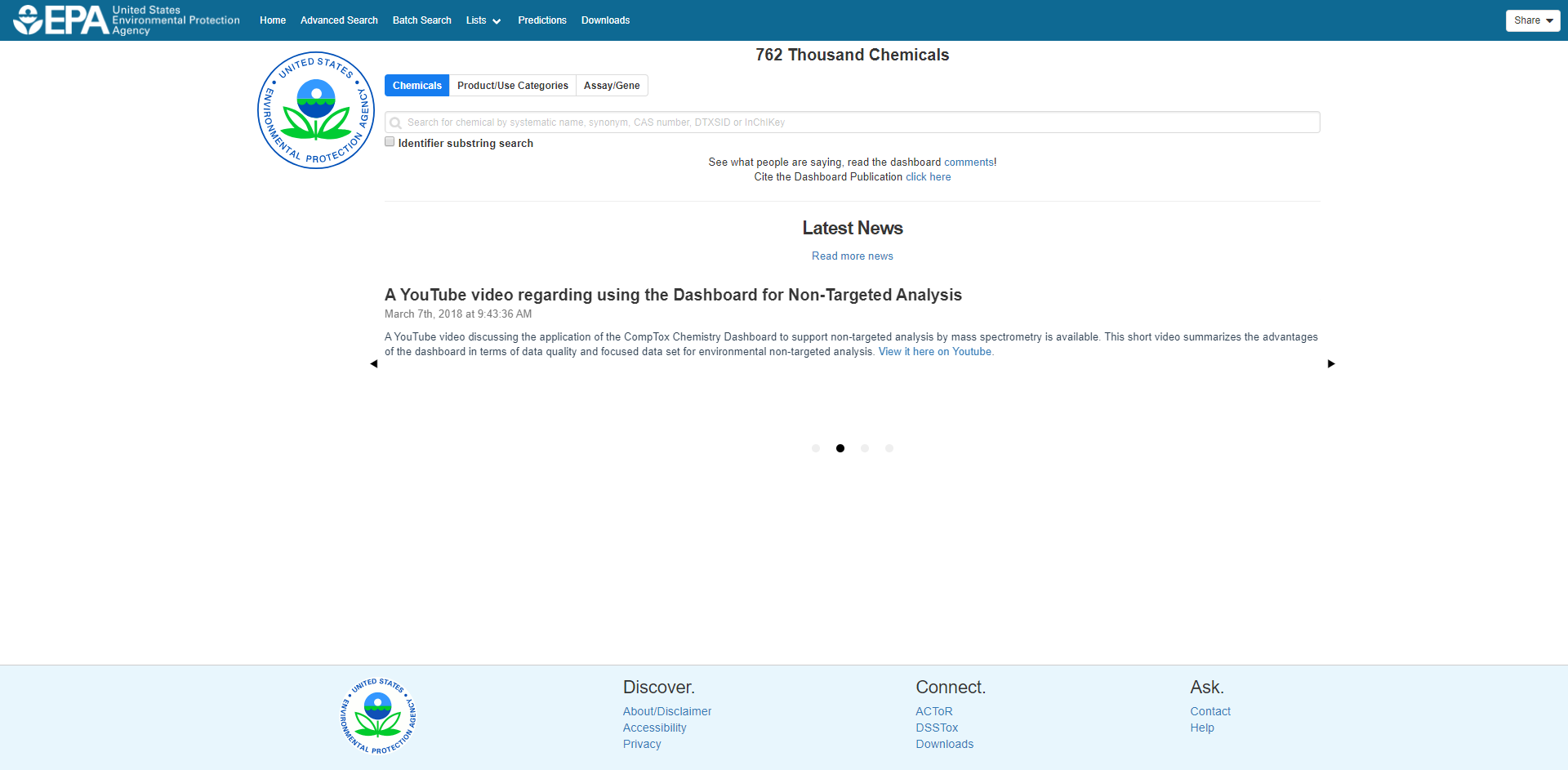


Figure S1. Homepage of the US EPA’s CompTox Chemistry Dashboard (<https://comptox.epa.gov>). Individual DTXSID pages can be accessed by entering a name, synonym, CAS number, or InChIKey. Data can also be queried from this page by using the Product/Use Categories and Assay/Gene search options. Advanced Search, Batch Search, and Lists can be accessed on the top left. Downloadable files can be accessed from the bottom panel under “Connect” and at the top under “Downloads”.


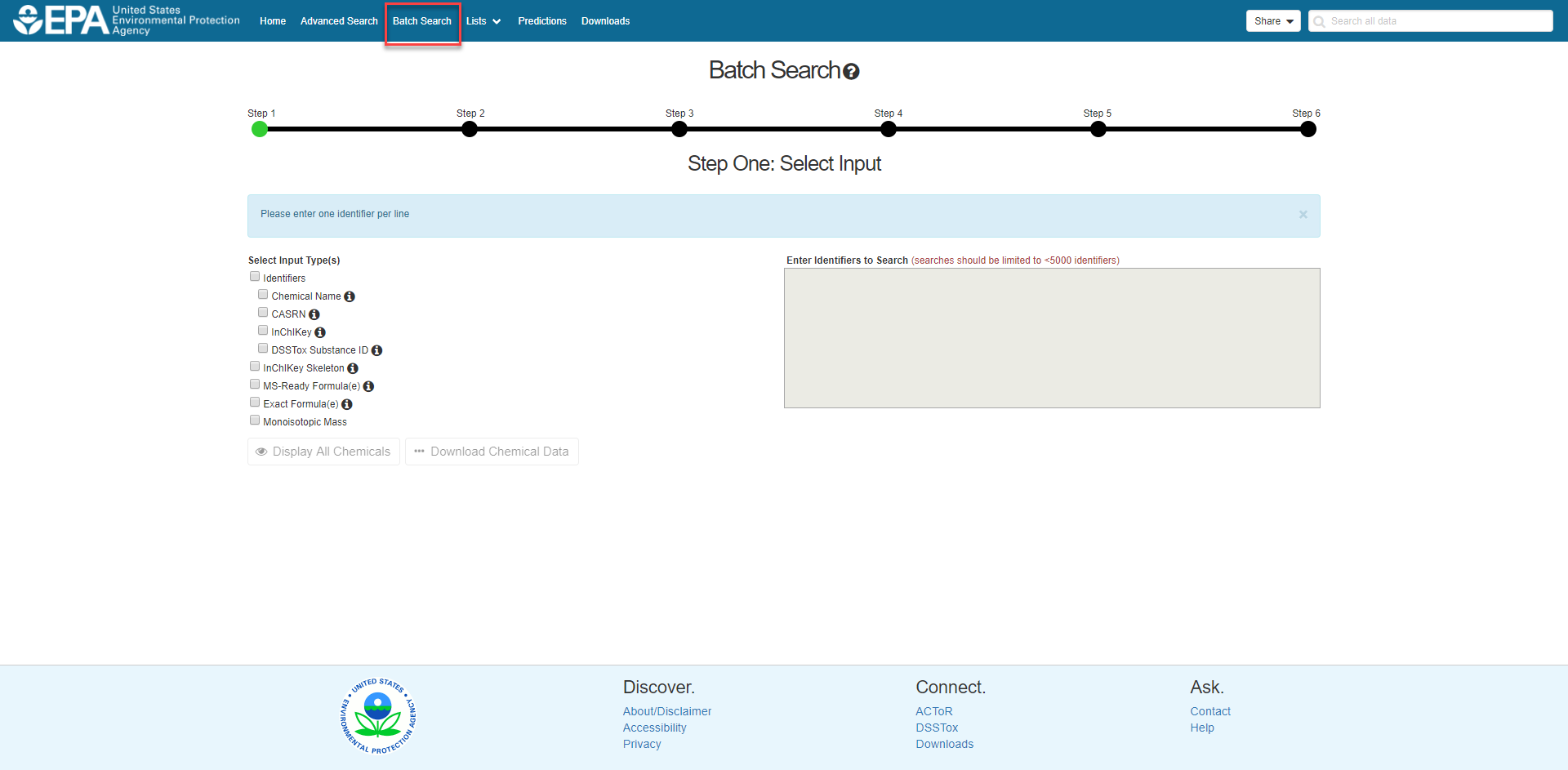


Figure S2. Batch Search page of the Chemistry Dashboard (<https://comptox.epa.gov/dashboard/dsstoxdb/batch_search>). Batches of chemical identifiers can be entered in the text box. MS-Ready Structures are searched when formulae are entered after selecting “MS-Ready Formula” or when “Monoisotopic Mass” are selected. A step-by-step indicator at the top walks users through the data input and download process.


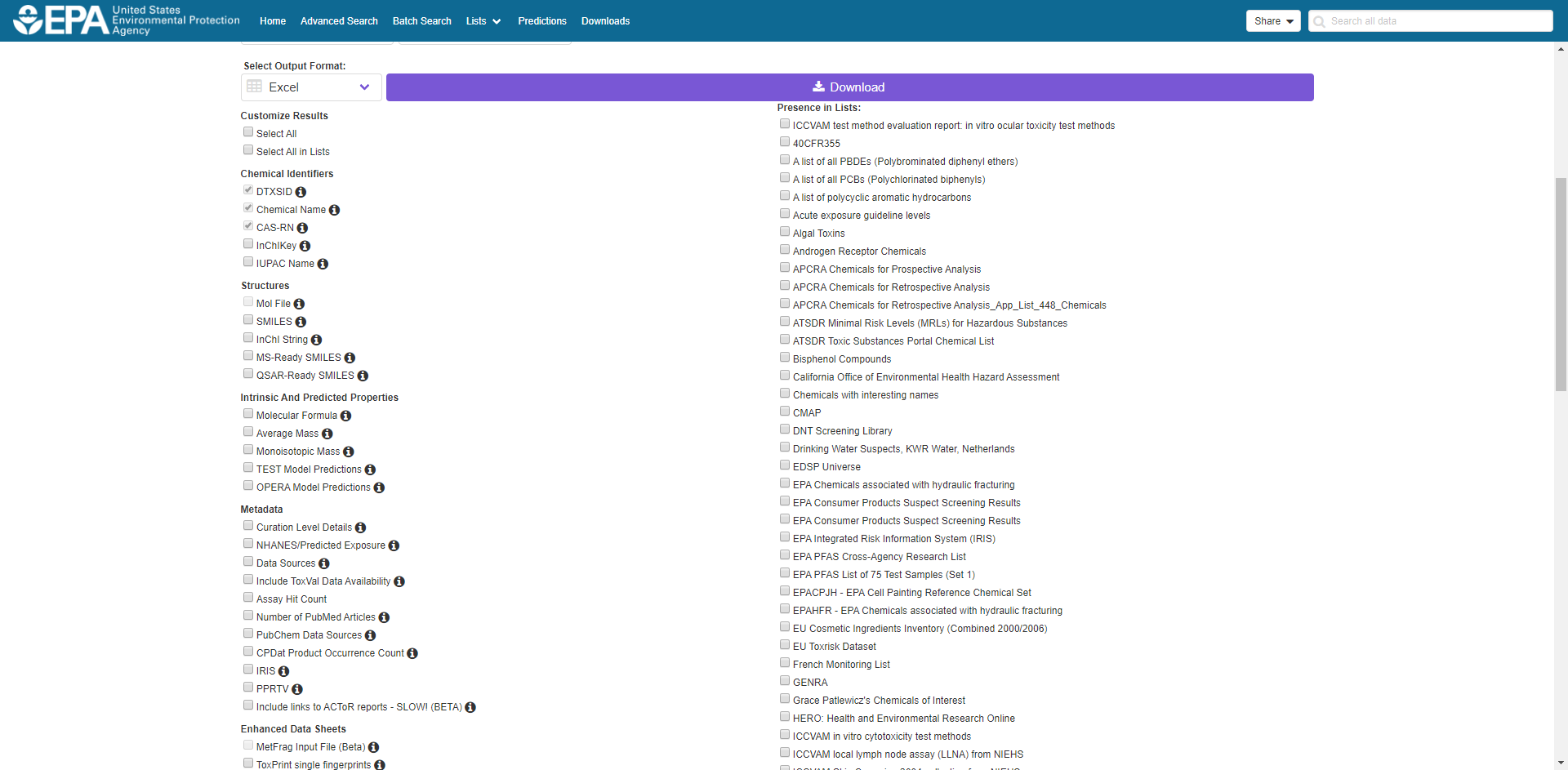


Figure S3. Batch Search download options. Once the input box is completed and output format is selected, additional data can be selected for inclusion in a download file. Data includes associated identifiers, structures (including MS-Ready SMILES), properties, metadata (data sources, exposure predictions, etc.) and presence in lists.


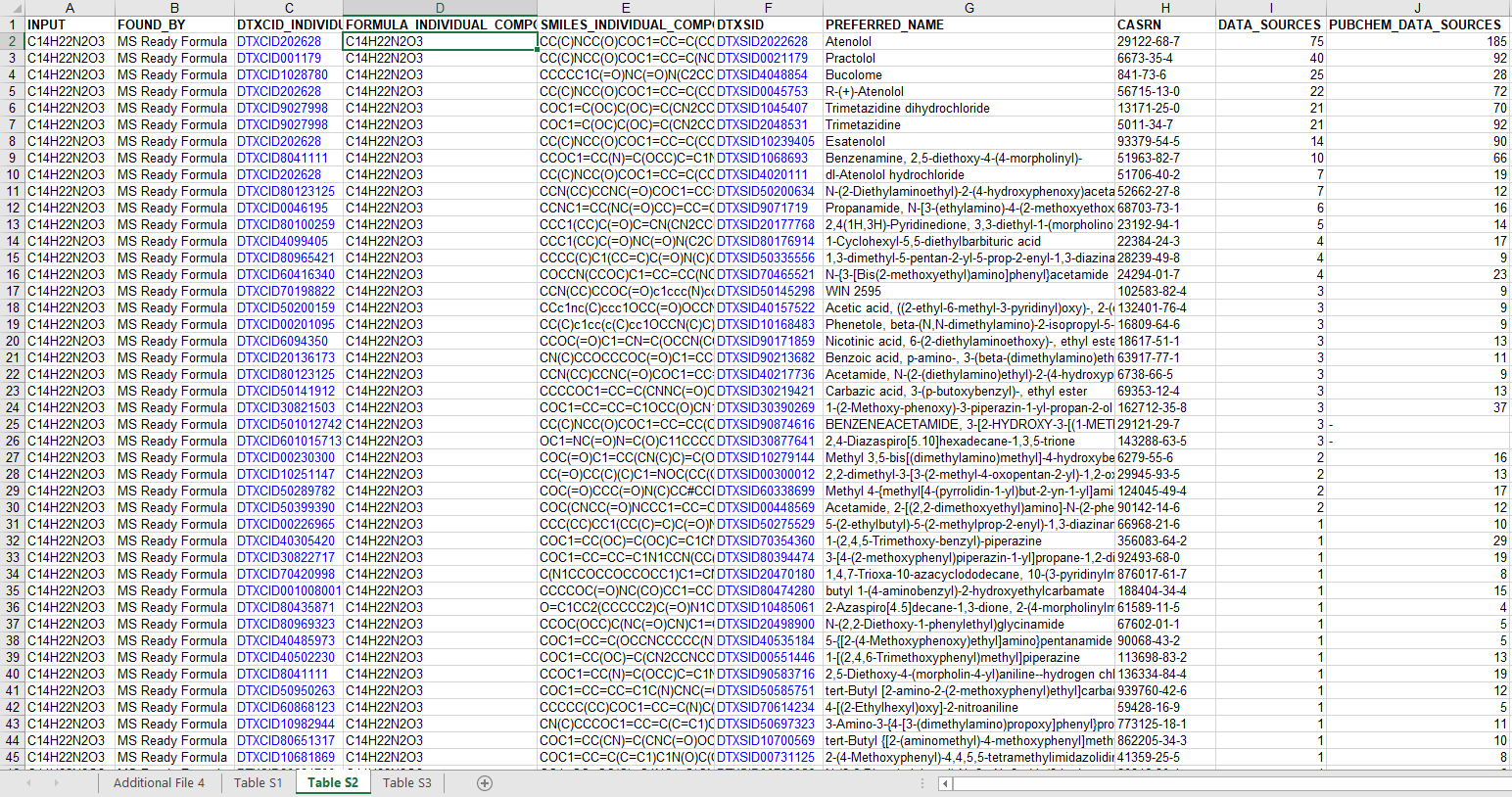


Figure S4. Results of a batch search download of C_14_H_22_N_2_O_3_ using MS-Ready Structures. Default columns are INPUT, FOUND_BY, DTXCID_INDIVIDUAL_COMPONENT, FORMULA_INDIVIDUAL_COMPONENT, SMILES_INDIVIDUAL_COMPONENT, DTSXID, and PREFERRED_NAME. CAS-RN, Data Sources, and PubChem Data Sources were selected for inclusion in the results.
