# Supplementary material for: “MS-Ready” structures for non-targeted high-resolution mass spectrometry screening studies
Source: J Cheminform. 2018 Aug 30;10:45. doi: 10.1186/s13321-018-0299-2 (PMC6117229; doi:10.1186/s13321-018-0299-2)
Supplement: Supplementary file 5 — Additional file 5. Additional MetFrag results and data (Figures S5–S8, Table S4). [file 13321_2018_299_MOESM5_ESM.docx]

Additional File 5, 4 Figures, 1 Table

“MS-Ready” structures for non-targeted high-resolution mass spectrometry screening studies

Andrew D. McEachran^1,2*^, Kamel Mansouri^1,2,3^, Chris Grulke^2^, Emma L. Schymanski^4^, Christoph Ruttkies^5^ and Antony J. Williams^2*^

^1^Oak Ridge Institute for Science and Education (ORISE) Research Participation Program, U.S. Environmental Protection Agency, 109 T.W. Alexander Dr., Research Triangle Park, NC 27711 USA

^2^National Center for Computational Toxicology, Office of Research and Development, U.S. Environmental Protection Agency, 109 T.W. Alexander Dr., Research Triangle Park, NC 27711 USA

^3^ Current address: Integrated Laboratory Systems, Inc., 601 Keystone Dr, Morrisville, NC 27650, USA

^4^Luxembourg Centre for Systems Biomedicine (LCSB), University of Luxembourg, 6, avenue du Swing, L-4367 Belvaux, Luxembourg.

^5^Department of Stress and Development Biology, Leibniz Institute of Plant Biochemistry (IPB), Weinberg 3, 06120 Halle (Saale), Germany.

Email addresses:

Kamel Mansouri: [kamel.mansouri@nih.gov](mailto:kamel.mansouri@nih.gov)

Chris Grulke: [grulke.chris@epa.gov](mailto:grulke.chris@epa.gov)

Emma L. Schymanski: [emma.schymanski@uni.lu](mailto:emma.schymanski@uni.lu)

Christoph Ruttkies: [christoph.ruttkies@ipb-halle.de](mailto:christoph.ruttkies@ipb-halle.de)

Andrew D. McEachran

[mceachran.andrew@epa.gov](mailto:mceachran.andrew@epa.gov)

Mail Drop D143-02

109 T.W. Alexander Dr.

Research Triangle Park, NC 27711 USA

Phone: 1-919-541-3001

Antony J. Williams

[williams.antony@epa.gov](mailto:williams.antony@epa.gov)

Mail Drop D143-02

109 T.W. Alexander Dr.

Research Triangle Park, NC 27711 USA

Phone: 1-919-541-1033


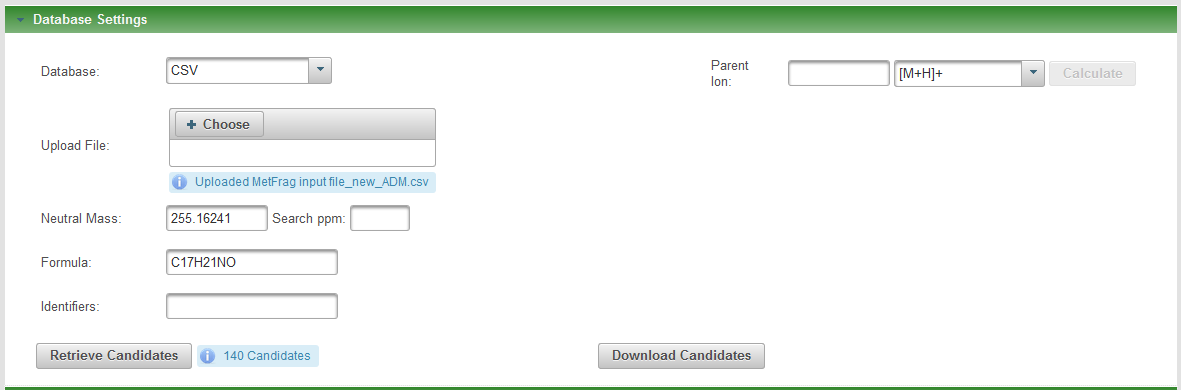

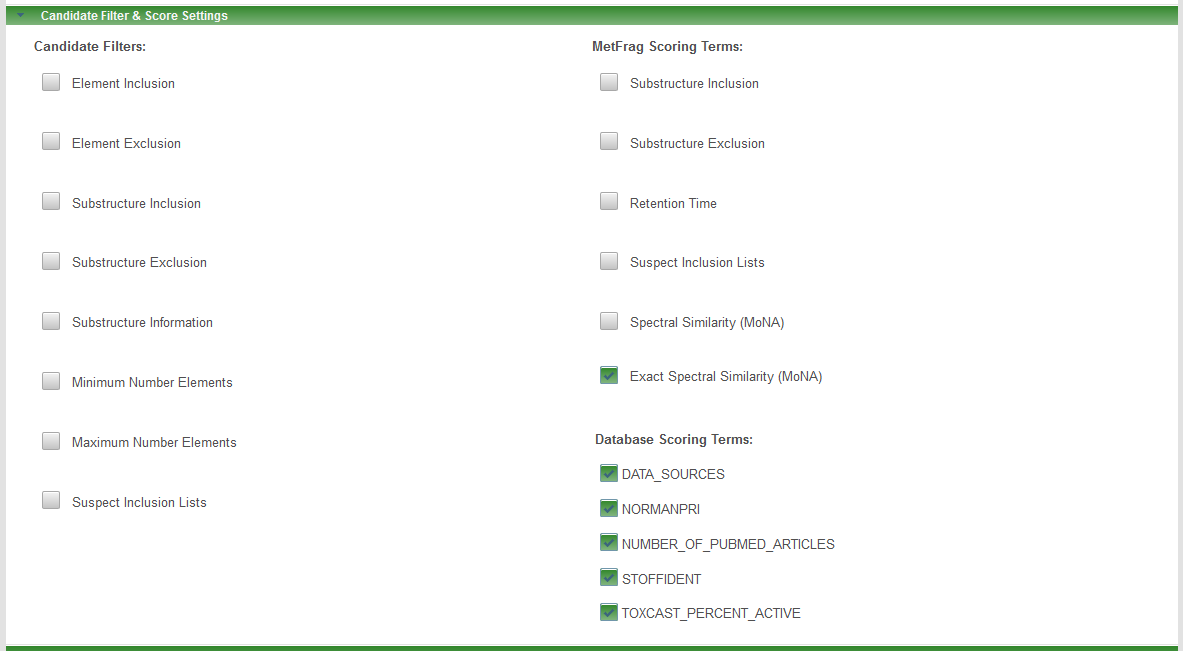


Figure S5: Automatic inclusion of additional Database Scoring Terms in MetFrag after upload of the CompTox Dashboard export file.

| 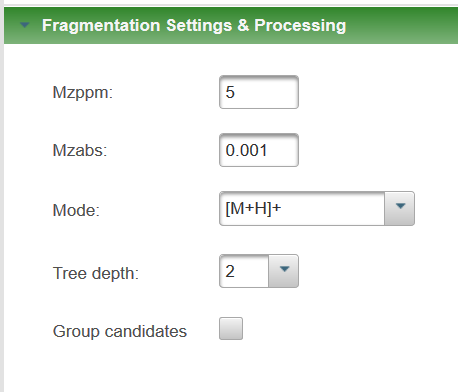 | 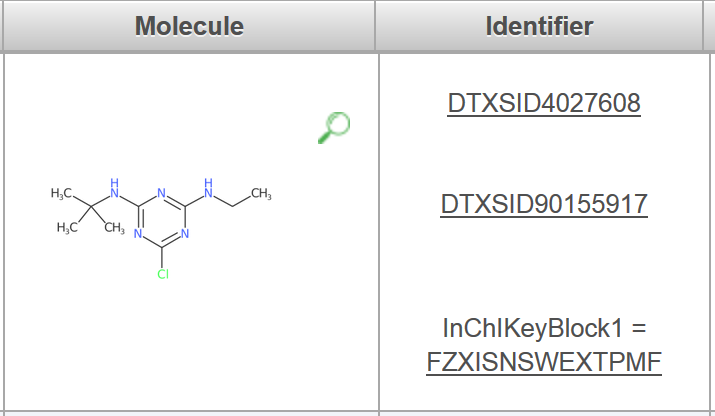 |
| --- | --- |

Figure S6. Left: The group candidate option (deselected). Right: Results with active “group candidate” option. The results from the top scoring candidate are presented, but all identifiers are displayed in the “identifier” window.


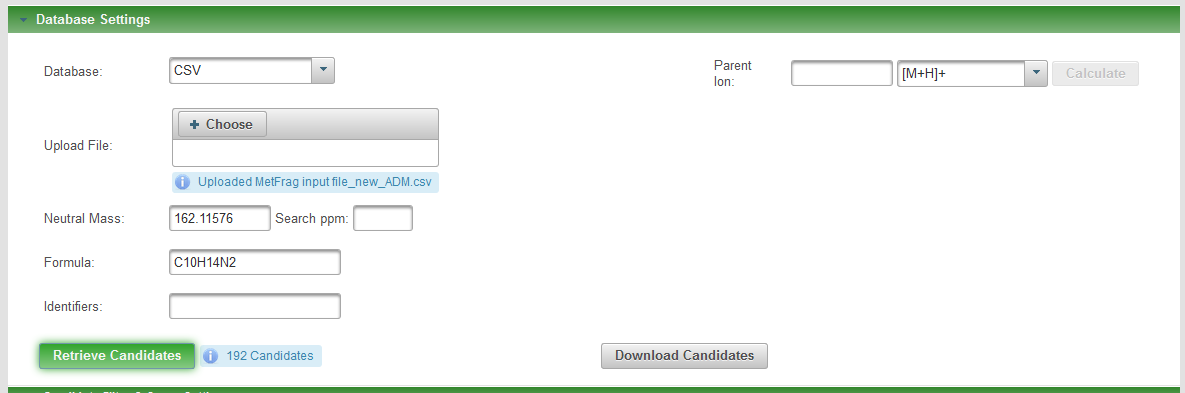


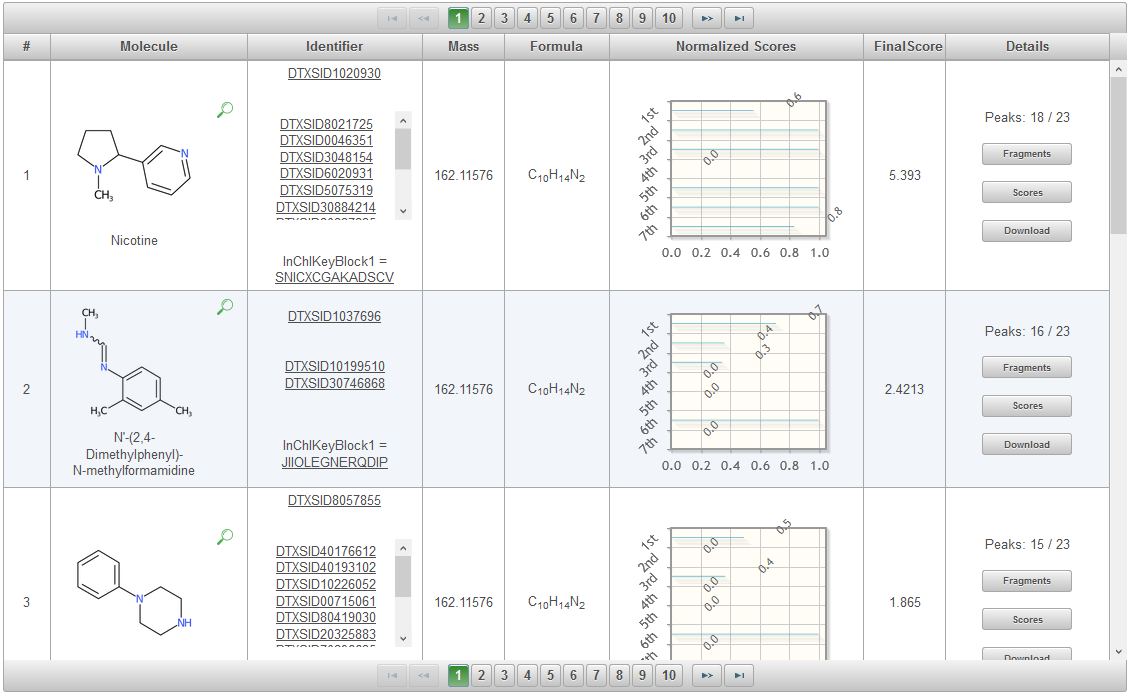


Figure S7. MetFrag input (top) and results (bottom) retrieved with the MS-Ready search for C10H14N2. The score categories are (1^st^ to 7^th^): MetFrag Fragmentation, Exact Spectral Similarity, Data Sources, Presence in NORMAN Priority list, Number of PubMed Articles, Presence in STOFF-IDENT, and Percent Active ToxCast Assays. Spectral data for Nicotine was retrieved from MassBank (<https://massbank.eu/MassBank/jsp/RecordDisplay.jsp?id=EQ300804&dsn=Eawag>). Nicotine has the highest score (5.39).


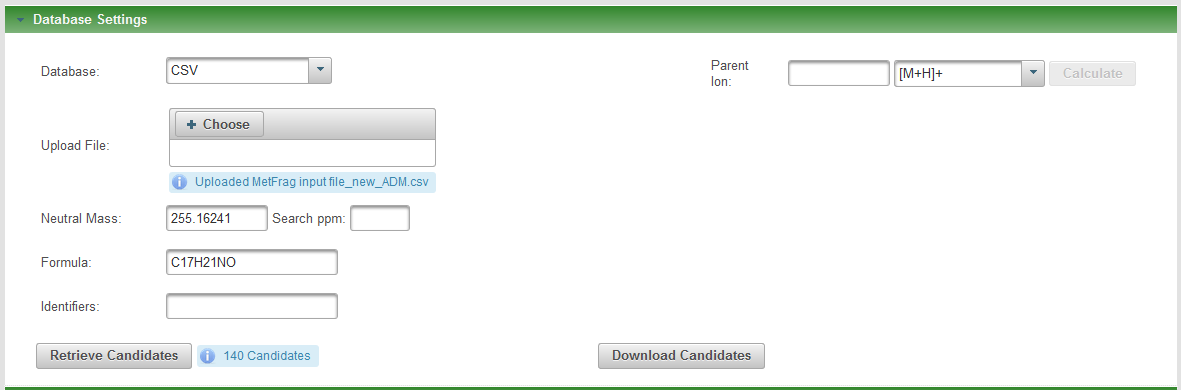


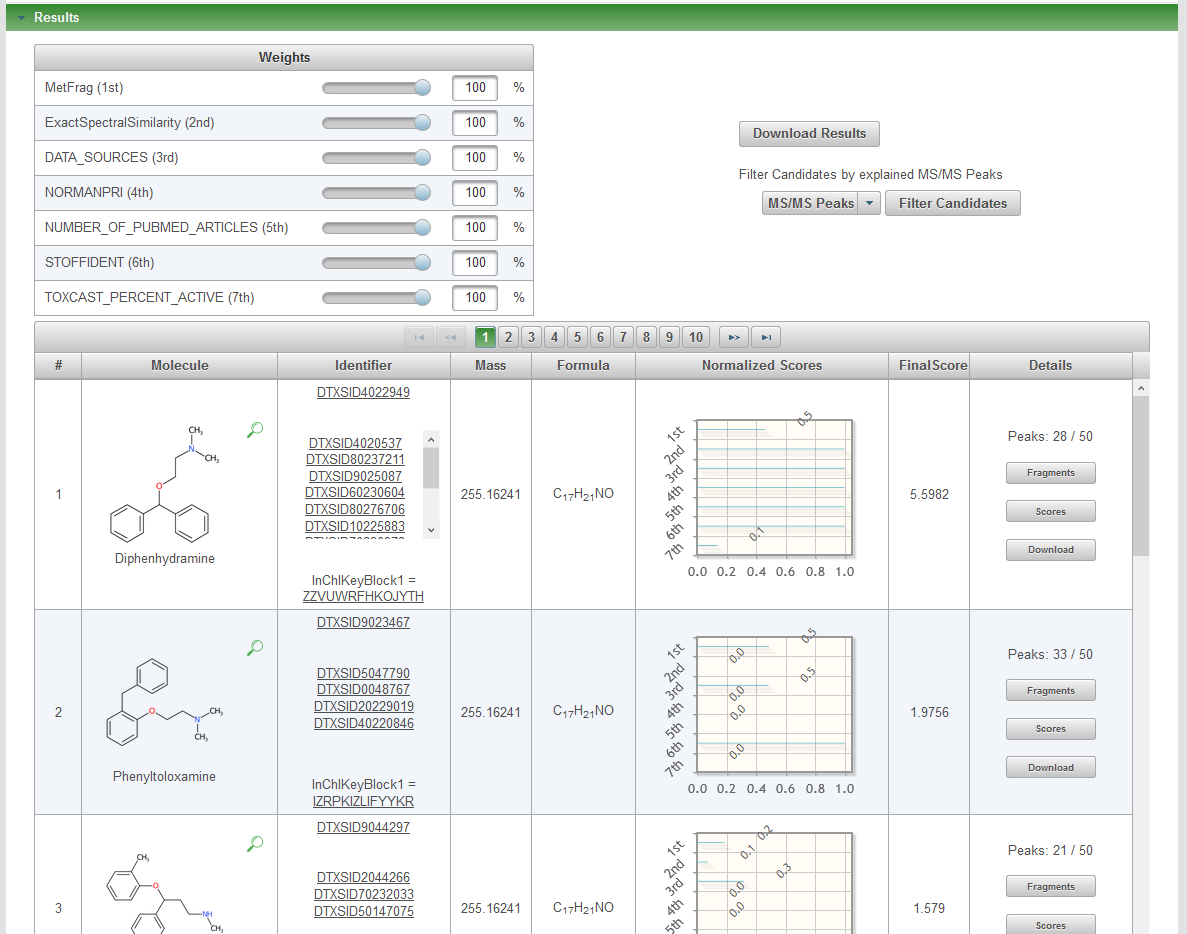


Figure S8. MetFrag input (top) and results (bottom) retrieved with the MS-Ready search for C17H21NO. The score categories are (1^st^ to 7^th^): MetFrag Fragmentation, Exact Spectral Similarity, Data Sources, Presence in NORMAN Priority list, Number of PubMed Articles, Presence in STOFF-IDENT, and Percent Active ToxCast Assays. Spectral data for Diphenhydramine was retrieved from MassBank (<https://massbank.eu/MassBank/jsp/RecordDisplay.jsp?id=EQ335208&dsn=Eawag>). Diphenhydramine has the highest score (5.60).

Table S4: MetFrag results for the query C_7_H_12_ClN_5_. The highest scoring entry for each field have been bolded. The highest total score is for Simazine, but the correct answer desethylterbutylazine is second and has the best spectral scores (MetFrag Raw Score, Exact MS Similarity).

| Identifier | Score | MetFrag Raw Score | # Expl  Peaks | Exact MS Similarity | Data Sources | NORMAN  Priority | PubMed Articles | Toxcast Active | STOFF Ident |
| --- | --- | --- | --- | --- | --- | --- | --- | --- | --- |
| DTXSID4021268 | **4.98** | 121.74 | 4 | 0.29 | **107.0** | 0.0 | **335.0** | **3.53** | **1.0** |
| DTXSID80184211 | 4.26 | **177.80** | **6** | **1.0** | 28.0 | **1.0** | NA | NA | **1.0** |
| DTXSID20407557 | 2.05 | **177.80** | **6** | 0.0 | 5.0 | 0.0 | NA | NA | **1.0** |
| DTXSID20190518 | 1.03 | **177.80** | **6** | 0.0 | 3.0 | 0.0 | NA | NA | 0.0 |
| DTXSID9062774 | 0.80 | 124.98 | 4 | 0.0 | 10.0 | 0.0 | NA | NA | 0.0 |
| DTXSID10192147 | 0.74 | 124.57 | 4 | 0.0 | 4.0 | 0.0 | NA | NA | 0.0 |
| DTXSID50615441 | 0.61 | 106.83 | 5 | 0.0 | 1.0 | 0.0 | NA | NA | 0.0 |
| DTXSID40527281 | 0.15 | 24.73 | 3 | 0.0 | 1.0 | 0.0 | NA | NA | 0.0 |
| DTXSID20585369 | 0.09 | 14.65 | 2 | 0.0 | 1.0 | 0.0 | NA | NA | 0.0 |
| DTXSID60337288 | 0.07 | 6.64 | 2 | 0.0 | 4.0 | 0.0 | NA | NA | 0.0 |
| DTXSID20500259 | 0.04 | 6.02 | 1 | 0.0 | 1.0 | 0.0 | NA | NA | 0.0 |
